# sTarPicker: A Method for Efficient Prediction of Bacterial sRNA Targets Based on a Two-Step Model for Hybridization

**DOI:** 10.1371/journal.pone.0022705

**Published:** 2011-07-22

**Authors:** Xiaomin Ying, Yuan Cao, Jiayao Wu, Qian Liu, Lei Cha, Wuju Li

**Affiliations:** 1 Center of Computational Biology, Beijing Institute of Basic Medical Sciences, Beijing, China; 2 Department of Clinical Laboratory, the 90th Hospital of Jinan, Shandong, China; J. Craig Venter Institute, United States of America

## Abstract

**Background:**

Bacterial sRNAs are a class of small regulatory RNAs involved in regulation of expression of a variety of genes. Most sRNAs act in trans via base-pairing with target mRNAs, leading to repression or activation of translation or mRNA degradation. To date, more than 1,000 sRNAs have been identified. However, direct targets have been identified for only approximately 50 of these sRNAs. Computational predictions can provide candidates for target validation, thereby increasing the speed of sRNA target identification. Although several methods have been developed, target prediction for bacterial sRNAs remains challenging.

**Results:**

Here, we propose a novel method for sRNA target prediction, termed sTarPicker, which was based on a two-step model for hybridization between an sRNA and an mRNA target. This method first selects stable duplexes after screening all possible duplexes between the sRNA and the potential mRNA target. Next, hybridization between the sRNA and the target is extended to span the entire binding site. Finally, quantitative predictions are produced with an ensemble classifier generated using machine-learning methods. In calculations to determine the hybridization energies of seed regions and binding regions, both thermodynamic stability and site accessibility of the sRNAs and targets were considered. Comparisons with the existing methods showed that sTarPicker performed best in both performance of target prediction and accuracy of the predicted binding sites.

**Conclusions:**

sTarPicker can predict bacterial sRNA targets with higher efficiency and determine the exact locations of the interactions with a higher accuracy than competing programs. sTarPicker is available at http://ccb.bmi.ac.cn/starpicker/.

## Introduction

Bacterial sRNAs are a class of small regulatory RNAs 40–500 nt in length. They are often encoded in the intergenic regions of bacterial chromosomes and generally untranslated [1]. To date, more than 1,000 sRNAs have been discovered in a variety of bacterial species, including *Escherichia coli*, *Salmonella,* and *Vibrio cholerae* [2]. These sRNAs play important roles in gene regulation, including regulation of biogenesis of outer membrane proteins [3], quorum sensing [4], [5], and expression of virulence-related genes [6], [7]. sRNAs function through base-pairing with mRNAs, binding proteins and altering their activity, and, in some cases, mimicking the structures of other RNA or DNA molecules (reviewed in [8]). Among these functional roles, base-pairing with mRNAs represents the major regulatory mechanism and can lead to translational repression, translational activation or mRNA degradation [9]. Regulation by a large number of base-pairing sRNAs characterized thus far requires the RNA chaperone protein Hfq. This protein is an Sm-like protein and conserved in a wide range of bacteria [10], [11].

In comparison to steady increases in the number of sRNAs identified, the number of characterized sRNA targets has only slowly increased. To our knowledge, distinct direct targets have been experimentally validated for only approximately 50 sRNAs [12]. The precise targets of most sRNAs remain elusive. Several experimental approaches have been applied to identify mRNA targets of sRNAs, including point mutation strategies, reporter gene assays, microarray profiling, and proteomics-based approaches. However, these experimental approaches have several shortcomings. First, although point mutation strategies and reporter gene assays can be used to identify direct targets, these approaches are more applicable to target validation, rather than *ab initio* target identification, due to the low-throughput nature of the assays. Second, although microarray and proteomics represent high-throughput approaches, they are only applicable to broad target screening, as these methods cannot distinguish direct targets from indirect targets. Further experiments are then necessary to validate targets after a small subset of candidate targets are obtained. Moreover, the microarray-based approach cannot detect targets that are translationally inhibited without degradation.

Computational prediction is a labor-saving methodology that is complementary to experimental approaches for sRNA target determination. Efficient prediction approaches can provide high quality candidates for target validation. To date, two groups of methods have been utilized in bacterial sRNA target prediction.

The first group is target prediction methods developed specifically for bacterial sRNAs. For example, Zhang et al. developed a method to predict the targets for Hfq-binding sRNAs in *E. coli* using a modified Smith-Waterman local sequence alignment algorithm [13]. Four characteristics were involved in this prediction method: (1) Hfq-binding sites, (2) sub-sequences ranging from –35 to +15 nt surrounding translation initiation sites in mRNAs, (3) candidates for loop or bulge regions in the sRNAs for alignment extension, and (4) conservation profiles of the sRNAs and their targets. The highest score and the corresponding alignment were selected as the final score and alignment for each sRNA-mRNA pair.

Tjaden et al. proposed a program for sRNA target prediction, termed TargetRNA, which included an individual base pair model and a stacked base pair model of hybridization scoring for sRNA-target interactions [14], [15]. The individual base pair model was based on a modified Smith-Waterman local sequence alignment algorithm. The stacked base pair model was a straightforward extension of RNA folding approaches with intra-molecular base-pairing prohibited. The *P*-value for an sRNA-target hybridization score was estimated with the distribution of ten thousand hybridization scores of the sRNA and random RNA sequences. The statistically significant sRNA-target interactions were selected as candidate interactions.

Mandin *et al*. proposed a method for sRNA target prediction by searching for the strong sRNA-mRNA duplexes [16]. Each sRNA-mRNA duplex was scored as a sum of both positive contributions, due to pairing nucleotides, and negative contributions, due to bulges and internal loops. The cost of bulges and internal loops were empirically gauged using four validated sRNA-mRNA interactions. Statistical significance of the duplex was used as the criterion for interaction, which was assessed by comparison to an ensemble of random sequences.

Our group has presented two models for target prediction using machine learning methods [17], [18]. Sub-sequences located within –30 to +30 nt of the start codons of targets were selected as core binding regions. Based on the hypothesis that sequences surrounding the core binding regions were also likely to influence the interactions, we extracted flanking sequences around the core binding regions using sliding windows. For each sub-sequence, ten features, including the percent composition of bases in interior loops, the minimum free energy (MFE) of hybridization, and the difference in the MFE values before and after hybridization, were taken into consideration. The models were then trained using the Tclass system [19] and support vector machines (SVM), respectively.

The second group is general RNA-RNA interaction prediction methods. These methods aimed to find the hybridization structure with the minimum binding energy for two RNA molecules. For example, inteRNA [20] was developed to minimize the joint free energy between the two RNA molecules under a number of energy models with growing complexity. This method allowed pseudo-knots in inter-molecular structures. RNAhybrid [21], [22] was a modification of the classic RNA secondary structure prediction, by neglecting intra-molecular base-pairings and multi-loops. This method was presented for miRNA target prediction, but it was also utilized in sRNA target prediction by Sharma et al. [23]. RNAplex [24] used a slightly different energy model to reduce computational time. Compared to RNAhybrid, RNAplex performed 10-27 times faster. RNAup [25] was designed to find the hybridization structure with the minimum extended hybridization energy which was the sum of hybridization energy and the energy necessary to make the binding sites accessible. Busch et al. proposed an approach, termed IntaRNA, which incorporated accessibility of binding sites of two RNA molecules, in addition to a user-definable seed [26]. Similar to RNAup, IntaRNA searched for the optimal interaction with the minimum extended hybridization energy, which was defined as the sum of the hybridization energy and the energy required to make the sRNA and target binding sites accessible. The MFE values for seed regions were involved in the calculation of the minimum extended hybridization energy.

Most of the above methods neglect the secondary structures of two RNA molecules before they interact. However, many authors have shown that target site accessibility is very important to target recognition for miRNAs and siRNAs [27]-[33]. The free energy alone of the hybridized duplex is a poor predictor for miRNA target prediction [34]. In fact, by comparisons using 18 validated sRNA-target interactions, Busch et al. showed that IntaRNA and RNAup, the only two methods that considered site accessibility, outperformed the other three methods that mainly considered the free energy of hybridized structures, namely TargetRNA, RNAhybrid and RNAplex. IntaRNA performed better than RNAup, probably because IntaRNA added the hybridization energy of seed for each candidate hybridized structure during the energy calculation.

Herein, we propose a novel method, sTarPicker, which is based on a two-step model of hybridization between an sRNA and an mRNA target. In the two-step model, the seed region of the sRNA first binds to the seed region of the target with perfect complementary base-pairing. Then, the initial hybrid elongates to form the complete sRNA-target interaction. We assumed that the hybrid of the seed regions formed in the first step should be stable enough to initiate the elongation of the interaction in the second step. Using this model, first, all possible duplexes between an sRNA and a target are screened and stable duplexes are selected. Next, the duplexes are extended to identify the entire binding sites by mimicking the second step of the model. Finally, an ensemble classifier was constructed to distinguish between true interactions and pseudo interactions using machine learning methods. In energy calculations for seed regions and binding regions, both thermodynamic stability and site accessibility of sRNAs and targets were considered.

## Methods

### Summary of the sTarPicker Program

**Figure 1:**
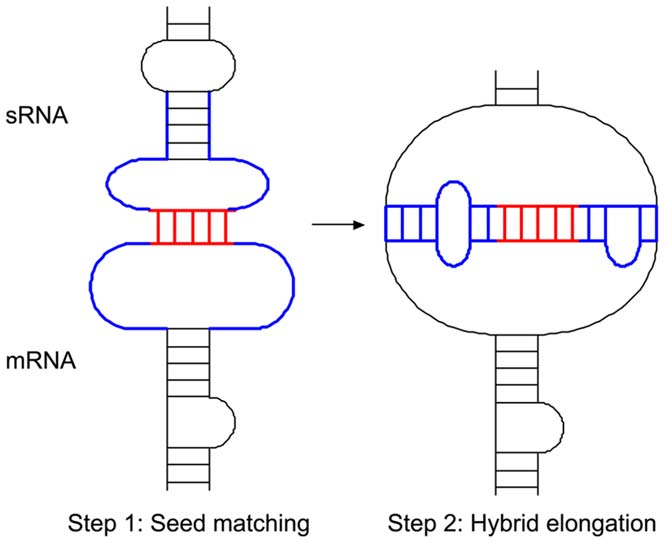
Two-step model for hybridization of sRNAs and targets.

We modeled hybridization of sRNAs and targets as a two-step process: (i) seed matching between the sRNA and a target, and (ii) elongation of the hybrid to form a stable inter-molecular duplex (Figure 1). Since both sRNAs and targets were fairly long, we considered both molecules to be structured in the process. Based on the two-step model for hybridization, sTarPicker gives prediction with the following four steps: (1) selects stable seeds from sRNA and putative target binding sequences, (2) extends binding sites from seed regions, (3) extracts features characterizing the binding sites, and (4) predicts with an ensemble classifier.

### Materials: Training and test datasets

To construct the classifier using machine learning methods, we retrieved 88 sRNA-mRNA pairs from our sRNATarBase release 1.0 [12]. These sRNA-mRNA pairs had been experimentally verified through techniques that included point mutation analysis and reporter gene assays. Four *cis*-encoded sRNAs and their targets were excluded. Among the remaining 84 *trans*-acting pairs, 76 pairs caused repression of the target mRNA (*i.e.* repression interaction), and 8 pairs caused activation of protein expression (*i.e.* activation interaction). To avoid bias within the training and test dataset, if multiple pairs in which the sRNAs and targets exhibited high levels of homology respectively (sequence similarity greater than 90%), only one pair were kept in the dataset. After removing redundant pairs, we obtained 54 unique repression pairs and 8 unique activation pairs.

Of the 54 unique repression pairs, 44 pairs included detailed binding sites, and 10 pairs included only the sRNA and target names. Of the 8 unique activation pairs, 7 pairs included detailed binding sites, and 1 pairs included only the sRNA and target name. Of the 51 pairs for which detailed binding sites were available, forty-nine of the target binding sites were located within −150 to +100 nt surrounding the start codon, including 96% of all unique pairs. Only one repressed target (ahrC of the SR1-ahrC pair) and one activated target (hla of the RNAIII-hla pair) bound sRNA outside of this region. Therefore, we selected sub-sequences between −150 and +100 nt of the start codon of the targets as the putative binding region for sRNAs. The reported sequence of the sRNA RybB in the RybB-ompW pair differed a little from RybB sequence retrieved from GenBank. Therefore, this pair was removed from the data. Finally, we obtained 42 unique repression pairs and 6 unique activation pairs with detailed binding sites. We randomly selected 32 of the 42 unique repression pairs as a training dataset (Table S1), and the remaining 10 pairs were used as positive test set.

During the course of our work, sRNATarBase deposited 7 newly found sRNA-mRNA repression pairs from release 1.0 to release 2.0, which had been experimentally verified to interact directly and the target binding sites were located within −150 to +100 nt surrounding the start codon. These repression pairs were non-redundant and were added to positive test set.

In total, seventeen unique repression pairs are used as positive test dataset, including 14 sRNAs distributed across six bacterial strains (Table S2).

To evaluate the specificities of the prediction methods, we constructed a non-interaction dataset as negative test dataset by randomly selecting target genes in genomes without replacement. For each sRNA in the positive test dataset, we randomly selected 10 target genes from the corresponding genome as non-interaction targets. Therefore, as 14 unique sRNAs were included in the positive test dataset, we obtained 140 non-interaction pairs in the negative test dataset.

### Computation of ΔG_hybrid_, ΔG_open_ and ΔΔG

Thermodynamic stability and site accessibility are two reliable indicators for hybridization between two RNA molecules. To assess the thermodynamic stability and site accessibility of seed regions and binding sites, we computed ΔG_hybrid_, ΔG_open_ and ΔΔG.

ΔG_hybrid_ was defined as the binding free energy of the hybridization structure of a specified sRNA region and a specified target region. ΔG_hybrid_ was computed using RNAduplex program from Vienna RNA package [35].

ΔG_open_ was defined as the energy required to make the specified region accessible for sRNA-target interactions. ΔG_open_ was computed as the difference between ΔG_unpaired_ and ΔG_paired_. ΔG_paired_ was defined as the free energy of the ensemble of all secondary structures of the specified region and additional flanking upstream and downstream sequences. ΔG_unpaired_ was defined as the free energy of the ensemble of all secondary structures of the specified region and additional flanking upstream and downstream sequences, in which the specified region was required to be unpaired. Full-length sequences were used for the calculations of ΔG_paired_ and ΔG_unpaired_ for sRNAs. For mRNA targets, the specified target region and 100 additional nucleotides of upstream and downstream flanking sequence were used to calculate ΔG_paired_ and ΔG_unpaired_. Flanking regions of 100 nucleotides in length were chosen based on the fact that there was a low probability of secondary structure base-pairing interactions between sequences separated by more than 100 nucleotides. For example, Lu and Mathews [36] found that over 75% of base pairs occurred between nucleotides separated by fewer than 100 nucleotides in rRNA with known secondary structure. Zhao et al. [37] and Kertesz et al. [28] used the value of 70 additional nucleotides of upstream and downstream for miRNA target prediction. Richter et al. [38] used 200 nt window for target prediction of sRNA Yfr1. Furthermore, this constraint also reduced significantly the time complexity of the computations described above. ΔG_paired_ was computed using RNAfold [35] with ‘-p0’ option, to calculate the ensemble free energy. ΔG_unpaired_ was computed using RNAfold with ‘-p0 -C’ options, to calculate the ensemble free energy when imposing the specified region to be single-stranded. In sRNA-target interactions, ΔG_open_ included both the energy of opening sRNA specified region and the energy of opening target specified region_._

ΔΔG was defined to be equal to the sum of ΔG_hybrid_ and ΔG_open_.

### Seed selection

For the two-step model of hybridization, stable hybridization of the seed region is a prerequisite for the second step. To select stable duplexes, we examined the secondary structures of the 32 experimentally-validated sRNA-mRNA pairs in the training dataset. We found that all of the corresponding secondary structures contained at least one duplex, with no less than five consecutive base pairs. The number of G-U and G-C base pairs within these duplexes was found to vary with duplex length. Shorter duplexes tended to have more G-C pairs and less G-U pairs. Based on these observations, potential helical regions that satisfied the following four conditions were kept for further analysis: (1) if the length of the helical region was 5 nt, G-U base pairs were forbidden and at least 4 G-C base pairs or 3 consecutive G-C base pairs must be present; (2) if the length of the helical region was 6∼7 nt, at most one G-U base pair and at least 3 G-C base pairs must be present; (3) if the length of the helical region was 8∼9 nt, at most one G-U base pair and at least 2 G-C base pairs must be present; and (4) if the length of the helical region was 10 nt or more, at most 4 G-U base pairs and at least 2 G-C base pairs must be present.

**Figure 2:**
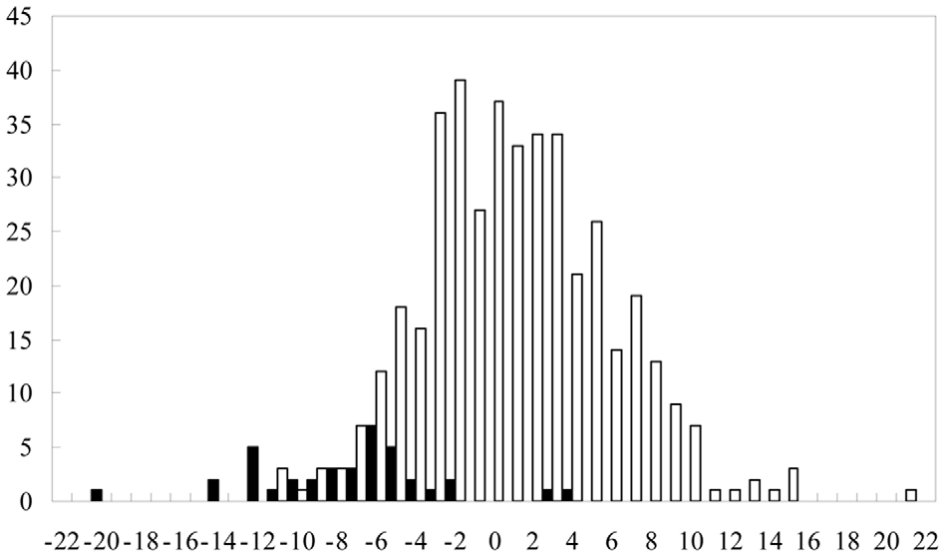
ΔΔG distributions for true and pseudo seed regions in the training dataset. ΔΔG includes contributions from both the hybridization energy and the energy required to make the sRNA and target binding sites accessible.

After applying the above restrictions in all the potential helical regions in the 32 training pairs, we obtained 460 potential seeds, including 38 true seeds (consistent with the reported hybrid structures) and 422 pseudo seeds (inconsistent with the reported hybrid structures). Among them, 38 true seeds were distributed in 32 training pairs. To examine the thermodynamic stability and site accessibility of these seeds, we calculated their ΔΔG values. Figure 2 shows the ΔΔG distribution of true seeds (in gray) and pseudo seeds (in white). From this distribution, it is evident that all but two true seeds had ΔΔG values less than −2 kcal/mol, whereas the majority of pseudo seeds had ΔΔG values greater than −2 kcal/mol. Therefore, based on these results, we selected −2 kcal/mol as the threshold for thermodynamic stability and site accessibility for seed regions. Using this constraint, we obtained 36 true seeds and 116 pseudo seeds. Among them, 36 true seeds were distributed in 31 training pairs. Therefore, using the seed selection criterion defined above, we recovered 97% (31/32) of the training pairs and only kept 27% (116/422) of the pseudo seeds.

To provide a more objective evaluation of the rationality of our seed selection method, we selected seeds between sRNAs and targets in the positive test data consisting of 17 unique pairs with detailed binding site information. Application of constraints for duplex length, number of G-C base pairs, and number of G-U base pairs resulted in the identification of 239 potential seeds, which included 24 true seeds and 215 pseudo seeds. Among them, 24 true seed were distributed in 16 test pairs. After further selection of seeds with ΔΔG values less than −2 kcal/mol, we obtained 70 potential seeds, including 20 true seeds and 50 pseudo seeds. Among them, 20 true seeds were distributed in 16 test pairs. Therefore, using this method, we recovered 94% (16/17) of the test pairs and only kept 23% (50/215) of the pseudo seeds. These percentages were similar to those derived from the training dataset.

Taken together, potential helical regions that satisfied the following five conditions were selected as candidate seeds: (1) if the length of the helical region was 5 nt, G-U base pairs were forbidden, and at least 4 G-C base pairs or 3 consecutive G-C base pairs must be present; (2) if the length of the helical region was 6∼7 nt, at most one G-U base pair and at least 3 G-C base pairs must be present; (3) if the length of the helical region was 8∼9 nt, at most one G-U base pair and at least 2 G-C base pairs must be present; (4) if the length of the helical region was 10 nt or more, at most 4 G-U base pairs and at least 2 G-C base pairs must be present; and (5) ΔΔG for the helical regions must be less than −2 kcal/mol. Potential helical regions between sRNAs and target genes were identified using GUUGle [39]. Terminal G-U base pairs were removed.

### Extension of binding sites from seed regions

The second step of the two-step model is the elongation of the interaction between the sRNA and the target. Full-length binding sites were obtained by extending potential interacting nucleotides on either side of the seed regions. We first computed a hybrid structure of the sRNA and the target sequence, imposing the sRNA seed to pair with the target seed. The target sequence included the target seed region and 100 additional nucleotides upstream and downstream. Next, we searched for interacting nucleotides on either side of the seed regions in the sRNA and the target sequence. If an intra-molecular base pair was encountered on one side, the search on this side was halted. Binding sites were defined as the maximum interacting regions in which no intra-molecular base pairs were formed. The hybrid structures were computed using RNAcofold [35] with ‘-C -noLP -noCloseGU’ parameters, to impose base-pairing of seed regions and to forbid lonely pairs and G-U base pairs at the end of helices. For the convenience of searching for full-length binding sites, the dot-bracket notations of the secondary structures were converted to ct files.

### Feature extraction

For each hybrid involving an sRNA binding site and a target binding site, we extracted 22 features describing the hybrid. The percentage of the nucleotides A, C, G, U, G+C, A+U, A+C in the hybrid were calculated as features 1-7, respectively. The percentage of paired nucleotides and the percentage of paired nucleotides in duplexes of at least 3 bp were defined as features 8 and 9. The ratio of the number of base pairs in duplexes of at least 3 bp to all base pairs was feature 10. The maximum number of consecutive base pairs was feature 11. The numbers of interior loops and bulges in the hybrid structure were features 12 and 13. The total number of interior loops and bulges was feature 14. The percentages of nucleotides in interior loops and bulges were features 15 and 16. The percentage of unpaired nucleotides was feature 17. The minimum free energy and ΔΔG of the hybrid were calculated as feature 18 and 19. The free energy and ΔΔG of the seed region were calculated as feature 20 and 21. The seed length was feature 22.

### Training of the ensemble classifier sTarPicker using the Tclass system

After selecting 36 true seeds and 116 pseudo seeds from the training dataset using the seed screening methods described above, we next extended sRNA and target binding sites beginning from these seeds. To avoid bias in the training data, we removed redundancies in the extended binding sites. For an sRNA-target pair, if multiple binding sites were consistent with experimentally-verified binding sites, only the site with the smallest ΔΔG value for the seed was selected as positive sample. For 116 binding sites extended from pseudo seeds, the binding sites that did not overlap with positive samples were selected as negative samples. Furthermore, if multiple negative samples overlapped with each other, only the site with the smallest ΔΔG value for the seed was selected as negative sample. In total, thirty-one binding sites were taken as positive samples and 102 non-redundant binding sites were taken as negative samples. The extended binding sites were compared with the corresponding validated binding sites reported in the literature. Results from this analysis revealed that the extended binding sites corresponded well to the validated binding sites (Table S3).

After obtaining positive and negative binding sites from the training dataset, we used the Tclass system [19] to select features and construct our classifier. The Tclass system integrates a wrapper method of feature-forward selection and ensemble classifier construction. This system was originally proposed for analysis of gene expression [19] and has been successfully applied to analysis of high-level expression of foreign genes in the pPIC9 vector [40] and to prediction of sRNA targets [17]. The number of features ranged from 1 to 10. For each number of features, ten subsets with the best leave-one-out cross-validation (LOOCV) classification accuracy were recorded.

**Figure 3:**
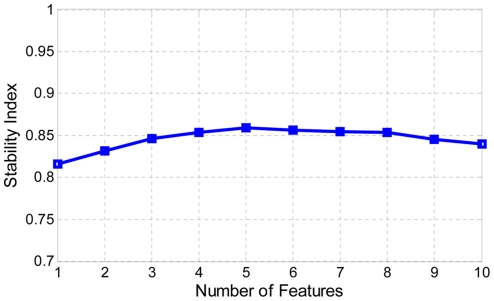
Variation of the stability index based on the number of features analyzed. The stability index represented the average of prediction accuracies over 1000 simulations with a training:test data partition ratio of 75%:25%.

To provide a more unbiased estimation, we performed stability analysis as follows. For each selected feature subset, we randomly partitioned 75% of the training dataset into a training subset for constructing classifier, and the remaining 25% of the dataset was used as the test subset for evaluating the performance of classifier. This process was repeated 1000 times, and the average classification accuracy from 1000 test subsets was defined as the stability index. Figure 3 shows the variation of the stability index according to the number of features. These results reveal that the highest stability index was obtained using five features. The feature subset with the highest stability index was chosen as the final feature subset. This subset included feature 1 (percentage of A nucleotides present in the hybrid), feature 4 (percentage of U nucleotides present in the hybrid), feature 18 (ΔG of the binding region), feature 19 (ΔΔG of the binding region) and feature 22 (seed length).

The corresponding 1000 classifiers generated as a part of the process of stability analysis were used as base classifiers for the final ensemble classifier, termed sTarPicker. Each base classifier gives a positive or negative prediction, indicating interacting or non-interacting respectively. The output of sTarPicker is the result of unweighted voting from 1000 base classifiers, that is, the ratio of the number of base classifiers giving positive predictions to the 1000 total classifiers. For example, if 500 base classifiers give positive predictions, the output score is 0.5. This value corresponds to the probability that the target interacts with the sRNA. Generally, if the probability is greater than 0.5, the target is considered to interact with the sRNA.

For sRNA target predictions, particularly genome-wide predictions of sRNA targets, high specificity is much more important than high sensitivity. This is due to the fact that high specificity reduces the number of false positives, providing a smaller number of candidate targets to be subjected to experimental validation. Therefore, to increase specificity, sTarPicker was trained with more negative samples than positive (negative : positive, 102 : 31). When sTarPicker was applied to the training dataset and the threshold probability was set to 0.5, the sensitivity and specificity were 54.84% (17/31) and 98.04% (100/102), respectively. When the threshold was set to 1, the sensitivity and specificity were 35.48% (11/31) and 99.02% (101/102), respectively.

### Comparisons with the existing methods

For comparative purposes, we also applied three state-of-the-art methods to the test dataset, namely IntaRNA [26], TargetRNA [14], [15], and sRNATarget [17], [18]. IntaRNA was chosen because this method was a typical general RNA-RNA interaction prediction method. Moreover, IntaRNA was demonstrated recently to outperform three other general RNA-RNA interaction prediction methods on 18 validated sRNA-target pairs. TargetRNA and sRNATarget were chosen because the methods were the only two available methods developed specifically for bacterial sRNAs.

For IntaRNA, sRNA target prediction was conducted under default parameter settings. Target regions used for IntaRNA ranged from −150 to +100 nt surrounding the start codon, identical to conditions for sTarPicker. Results from IntaRNA were sorted in ascending order of the energies.

For TargetRNA, we used the available web service with two groups of parameter settings. One group of parameters was the optimal settings that Tjaden et al. obtained in their paper [14]. The other group of parameters was identical to the parameters used in sTarPicker. To distinguish between the results of the two parameter settings, we denoted the method with the default parameter settings by TargetRNA and the method with the other parameter settings by TargetRNA2. If a target was identified in the list of the predicted candidates, the *P*-value was recorded. In all other cases, sRNA-target pairs were considered to be non-interaction predicted by TargetRNA. The results were sorted in ascending order of the *P*-values.

For sRNATarget, we used a local program under default parameters. Target regions ranged from −30 to +30 nt surrounding the start codon, which was the best interval in sRNATarget [17]. To eliminate the bias introduced by different training samples and different target regions, we re-trained the model with the 32 sRNA-target pairs from sTarPicker as positive training samples and 64 non-interaction pairs randomly selected from sRNATarBase [12] as negative samples. The core binding regions were sub-sequences from −150 to +100 nt surrounding the start codon and the flanking regions were sub-sequences from −200 to +120 nt surrounding the start codon. The new model was denoted by sRNATarget2. The results were sorted in descending order of the sRNATarget scores.

We employed sensitivity and positive predictive value (PPV) to compare the prediction accuracy of binding sites. Sensitivity and PPV were calculated as follows:
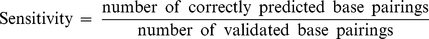


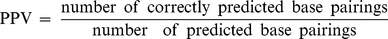



These measures have also been used to compare the prediction accuracy of several target prediction methods in IntaRNA [26].

### Web application design

To provide a convenient tool for users, we implemented a web application for sTarPicker. Two versions were designed for the distinct purposes of genome-wide target prediction and evaluation of interactions.

The genome-wide target prediction application can be used to examine all possible targets for an sRNA among all protein-coding genes in the genome. The search is executed after users input the following four parameters: bacterial genome, sRNA sequence, threshold for sTarPicker probability, and user email address. At the completion of the job, sTarPicker will notify the user via an email. The output includes all of the candidate target genes and the corresponding sTarPicker probabilities, ΔΔG values for binding regions and seed regions, and the secondary structures of the interactions. Candidate target genes are sorted based on sTarPicker probability (descending), followed by ΔΔG of target regions (ascending), and finally by ΔΔG of seed regions (ascending). If an sRNA-target pair returns multiple candidate interactions, all are listed.

The interaction evaluation tool can be used to assess the interaction between selected sRNAs and targets. The program is executed after users input the following six parameters: bacterial genome, sRNA name and sequence, sRNA mutation information, target gene name and sequence (or genomic locus), target mutation information, and threshold for sTarPicker probability. sRNA and target mutation information are optional, but these options are particularly useful when users are interested in analyzing the interaction between an sRNA and a target for which either or both sequences have been subjected to mutational analysis. Results are returned instantly and include all candidate interactions between the sRNA and the target, the corresponding sTarPicker probabilities, ΔΔG values for binding and seed regions, and secondary structures of the interactions.

## Results

### Performance on the test dataset

To evaluate the performance of sTarPicker, we applied the program to our test dataset, which included 17 interaction pairs and 140 non-interaction pairs. The target region was defined as a sub-sequence containing 150 nt upstream and 100 nt downstream of the first base of the start codon. The interval [−150, +100] was determined because the target binding sites of 96% of validated sRNA-target pairs located in the interval. When the threshold for sTarPicker probability was set to 0.5, the sensitivity and specificity were 76.47% (13/17) and 99.29% (139/140), respectively. When the threshold was set to 1, the sensitivity and specificity were 35.29% (6/17) and 100% (140/140), respectively. In order to generate a ROC curve (receiver operator curve) for sTarPicker, the results were sorted based on sTarPicker probability (descending), followed by ΔΔG of target regions (ascending), and finally by ΔΔG of seed regions (ascending). If an sRNA-target pair had several predicted binding regions, the region with the smallest rank was selected.

To compare with the existing methods, we applied three state-of-the-art methods to the test dataset, namely IntaRNA, TargetRNA, and sRNATarget. IntaRNA performance was evaluated using 17 interactions and 140 non-interactions, identical to the analysis of sTarPicker performance. TargetRNA performance was evaluated with two groups of parameter settings. The results of the two groups of parameter settings were denoted by TargetRNA and TargetRNA2, respectively. For TargetRNA and TargetRNA2 assessment, interaction Qrr1-luxR and ten non-interactions involving the sRNA Qrr1 in *Vibrio harveyi* were excluded because the web service of TargetRNA did not provide the corresponding genome. Two interactions, MicF-ompF and GcvB-oppA, were excluded since they were used as training samples in the method of TargetRNA. Ten non-interactions involving the sRNA MicF were also excluded due to the absence of MicF after the exclusion of interaction MicF-ompF. Therefore, the performances of TargetRNA and TargetRNA2 were evaluated using 14 interactions and 120 non-interactions. sRNATarget performance was evaluated with the published model [18] and the re-trained model with the same training dataset and target regions from sTarPicker. For analysis of sRNATarget, two interactions, MicF-ompF and GcvB-oppA, were excluded since they were used as training samples. Ten non-interactions involving the sRNA MicF were also excluded from the test dataset. Therefore, sRNATarget performance was evaluated using a total of 15 interactions and 130 non-interactions. sRNATarget2 performance was evaluated using 17 interactions and 140 non-interactions, identical to the analysis of sTarPicker performance.

**Figure 4:**
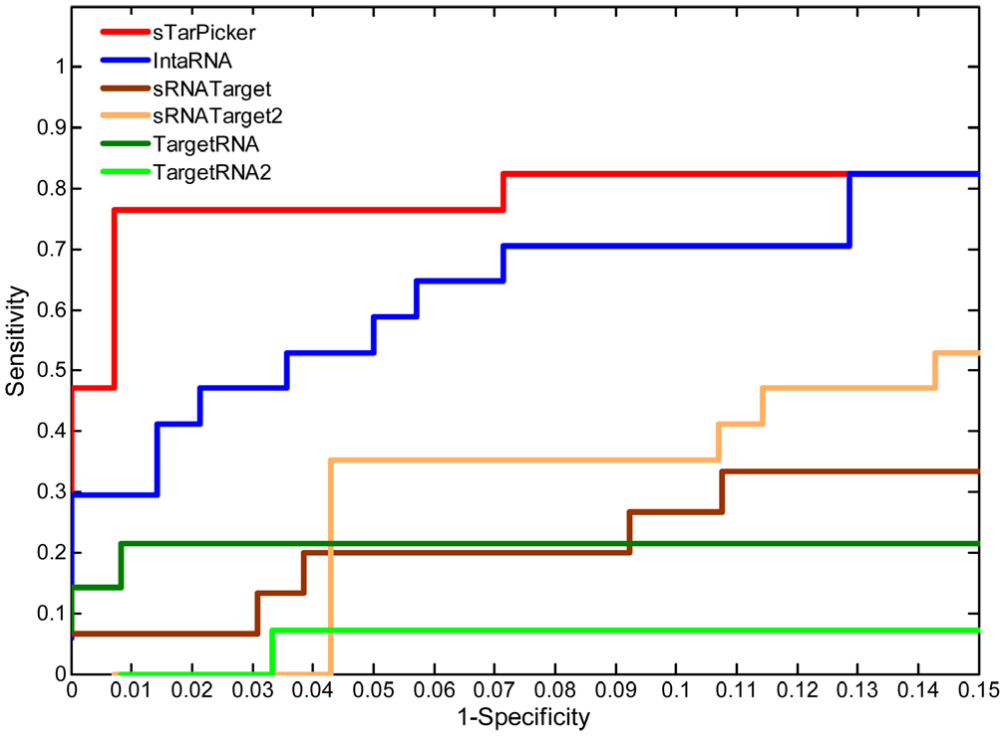
ROC curves of sTarPicker and three state-of-the-art prediction methods on the test dataset. Results from sTarPicker were sorted based on sTarPicker probability (descending), ΔΔG of binding regions (ascending), and ΔΔG of seed regions (ascending). If an sRNA-target pair returned several binding sites, only the site with the smallest rank was selected. Results from IntaRNA were sorted in ascending order of the energies. Results from sRNATarget and sRNATarget2 were sorted in descending order of the prediction scores. Results from TargetRNA and TargetRNA2 were sorted in ascending order of the *P*-values. The ROC curve was generated from the rates of true and false predictions while varying the number of considered interactions in all of the test data.

Figure 4 shows the ROC curves for the four methods applied to the test dataset. Based on these curves, it is evident that sTarPicker performed best, followed by IntaRNA. TargetRNA, TargetRNA2, sRNATarget and sRNATarget2 performed the third. Taking into consideration that TargetRNA and TargetRNA2 limited the number of reported candidate targets, the performance of TargetRNA and TargetRNA2 was conserved.

We next further compared the prediction accuracy of binding sites by sTarPicker, IntaRNA and TargetRNA. Table 1 shows a comparison of predictions for 17 sRNA-target pairs from the test dataset with detailed binding site information available. sRNATarget was not included in the comparison because it does not provide binding sites.

**Table 1:** Prediction accuracy of binding sites on seventeen validated sRNA-target pairs.

sRNA-target	Sensitivity				PPV			
	sTarPicker	IntaRNA	TargetRNA	TargetRNA2	sTarPicker	IntaRNA	TargetRNA	TargetRNA2
GcvB-sstT	0.417	0.000	-	-	0.172	0.000	-	-
MicF-ompF	0.840	1.000	0.560*	0.560*	1.000	1.000	0.636*	0.636*
MicA-phoP	0.455	0.455	1.000	-	1.000	1.000	1.000	-
OmrA-csgD	0.737	0.684	-	-	1.000	1.000	-	-
GcvB-livK	0.625	0.625	-	-	0.652	0.652	-	-
GcvB-oppA	0.913	1.000	1.000*	-	0.955	1.000	1.000*	-
InvR-nmpC	0.927	0.000	-	-	0.704	0.000	-	-
MicA-lamB	1.000	1.000	-	-	0.885	0.821	-	-
MicA-ompX	1.000	0.900	-	1.000	0.714	1.000	-	0.769
RybB-ompN	1.000	1.000	1.000	-	1.000	1.000	1.000	-
MicC-nmpC	1.000	1.000	-	-	1.000	1.000	-	-
PrrF1-sodB	0.840	0.600	-	-	1.000	0.789	-	-
Qrr1-hapR	0.708	0.708	-	-	1.000	1.000	-	-
Qrr1-luxO	0.444	0.444	-	-	1.000	1.000	-	-
MicX-VC0620	0.406	0.938	0.969	-	0.765	1.000	0.969	-
Qrr1-luxR	-	0.000	-	-	-	0.000	-	-
LhrA-lmo0850	1.000	0.786	-	-	0.824	1.000	-	-
Average on 17 pairs	**0.724**	0.655	0.266	0.092	**0.804**	0.780	0.271	0.083

*Indicates that the interaction was in the training dataset. -Indicates that no interaction was predicted. The highest average sensitivity and PPV are shown in bold.

As shown in Table 1, sTarPicker outperformed IntaRNA, TargetRNA and TargetRNA2 in the prediction accuracy of binding sites. IntaRNA achieved the second best. TargetRNA achieved the third and TargetRNA2 achieved the fourth. sTarPicker missed interaction Qrr1-luxR due to the absence of seed in length of 5 nt or more. However, sTarPicker still achieved the best averaged value of sensitivity and PPV among the three methods. IntaRNA reported putative binding sites for all 17 pairs, but three of them were completely different from the binding sites validated by experiments. The accuracies of TargetRNA and TargetRNA2 were far low because this method only reported 5 or 2 out of 17 pairs due to the cutoff of *P* value. The detailed information was provided in Table S4.

Although repression and activation of translation might exhibit different interaction characteristic, it is undertaken that the isolation of ribosome binding sites (RBS) on the target mRNAs after sRNAs bind targets causes translational repression, and the exposure of RBSs that are sequestered by structures of mRNAs themselves causes translational activation [41]. To evaluate the performance of sTarPicker on activation pairs, we applied sTarPicker on the six unique activation pairs with target binding sites within the interval [−150,+100] surrounding the start codon. When the threshold for sTarPicker probability was set to 0.5 and 1, the sensitivities were 50.00% (3/6) and 33.33% (2/6) respectively (Table S5). The performance was similar to those derived from the repression pairs in training and test datasets. This result suggests that sTarPicker can also be used to predict or evaluate the interactions that cause translational activation, despite the fact that this method was trained with repression interactions.

### Performance in genome-wide predictions of sRNA targets

To evaluate the ability of sTarPicker to identify sRNA targets genome-wide, we used sTarPicker to predict sRNA targets for 14 sRNAs from 6 bacterial strains in the test dataset. The genome sequences and annotation of protein-coding genes were downloaded from GenBank database of the National Center for Biotechnology Information [42]. For each sRNA, we extracted target regions ranging from −150 to +100 nt surrounding the start codon for all annotated genes in the corresponding genome. In total, 60,713 interactions were obtained (Table S2), including 16 true interactions from the test dataset and 7 true interactions from the training dataset. The interaction of Qrr1-hapR was excluded because the target gene hapR was not annotated in the genome of *Vibrio cholerae* obtained from GenBank. The remaining 60,690 interactions were all considered non-interactions. Results for each sRNA were sorted based on sTarPicker probability (descending), followed by ΔΔG of binding regions (ascending), and finally by ΔΔG of seed regions (ascending). If an sRNA-target pair returned several binding sites, only the binding site with the highest rank was selected.

In addition to sTarPicker, we also used IntaRNA, TargetRNA and sRNATarget for genome-wide predictions of sRNA targets for the same 14 sRNAs with the following exceptions. The parameter settings and putative target binding regions for the three methods were identical to the settings and putative binding regions described in Methods part. For IntaRNA, performance was evaluated using 16 true interactions and 60,690 non-interactions, identical to sTarPicker. For TargetRNA and TargetRNA2, the sRNA Qrr1 in *Vibrio harveyi* was excluded as the web service of TargetRNA did not provide the corresponding genome. Two interactions, MicF-ompF and GcvB-oppA, were excluded since they were used as training samples for the method of TargetRNA. The sRNA MicF was also excluded due to its absence after the exclusion of the two interactions. Therefore, this method was analyzed with a total of 50,644 interactions from 12 sRNAs distributed in five genomes, including 13 true interactions from the test dataset and 8 true interactions from the training dataset. The remaining 50,623 interactions were considered to be non-interactions. For assessment of sRNATarget, we excluded two interactions, MicF-ompF and GcvB-oppA, from our test dataset, since they were included in the training data for sRNATarget. The sRNA MicF was also excluded due to its absence after exclusion of the two interactions. Therefore, this method was analyzed with a total of 56,564 interactions from 13 sRNAs distributed in six genomes, including 14 true interactions from the test dataset and 8 true interactions from the training dataset. The remaining 56,542 interactions were considered to be non-interactions. For sRNATarget2, performance was evaluated using 16 true interactions and 60,690 non-interactions, identical to sTarPicker.

**Figure 5:**
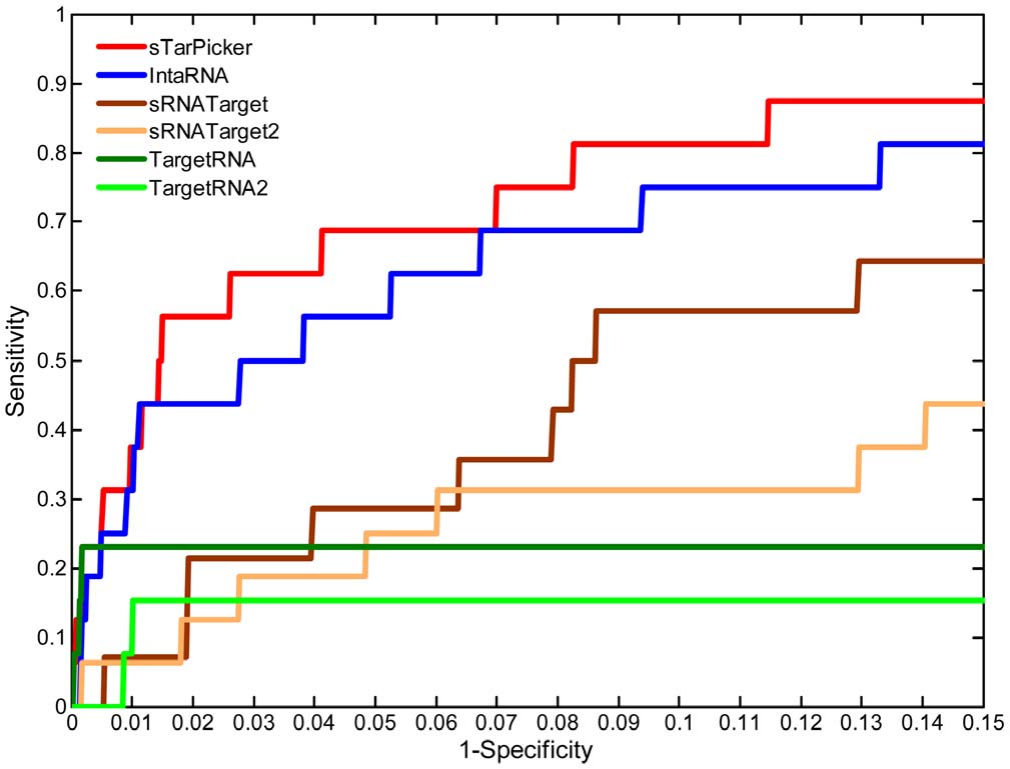
ROC curves of sTarPicker and three state-of-the-art methods in genome-wide prediction of sRNA targets. For sTarPicker, the results for each sRNA were sorted based on sTarPicker probability (descending), ΔΔG of binding regions (ascending), and ΔΔG of seed regions (ascending). If an sRNA-target pair returned several binding sites, only the site with the smallest rank was selected. For IntaRNA, results for each sRNA were sorted in ascending order of the energies. For sRNATarget and sRNATarget2, results for each sRNA were sorted in descending order of the prediction scores. For TargetRNA and TargetRNA2, results for each sRNA were sorted in ascending order of the *P*-values. Each ROC curve was generated by calculating sensitivity and specificity while varying the number of predicted interactions taken into consideration for each sRNA.

Figure 5 shows the ROC curves for all four methods on genome-wide target predictions of 14 sRNAs. Each ROC curve was generated by calculating sensitivity and specificity while varying the number of predicted interactions taken into consideration for each sRNA. Based on ROC curve analysis, it is evident that sTarPicker exhibited the best performance, better than IntaRNA, sRNATarget, sRNATarget2, TargetRNA and TargetRNA2. TargetRNA exhibited the best performance when the false positive rate was less than 0.005. However, TargetRNA only reported 3 out of 13 true interactions evaluated. It is worth noting that all positive predictions excluding true interactions from the training and test datasets were classified as false positives. However, some of these false positives may indeed represent actual interactions. Therefore, the estimated sensitivity and specificity values for all four methods may in fact be conservative. In particular, estimates of TargetRNA and TargetRNA2 performance may be more conservative, as this method only provides targets with *P* value less than 0.01 for each sRNA.

### A case study: target prediction of the sRNA Yfr1 in *Prochlorococcus*

During the time in which sTarPicker was being developed, Richter et al. published a study in which IntaRNA was used to discover two novel targets for the sRNA Yfr1 in *Prochlorococcus* MED4 [38]. Therefore, to compare sTarPicker with IntaRNA, we applied sTarPicker to the prediction of Yfr1 targets in the *Prochlorococcus* MED4 genome (Genbank accession number NC_005072). The putative target regions included sequences within −150 to +100 nt of the start codon for all annotated genes. We also utilized sRNATarget to predict targets of Yfr1. Since TargetRNA does not provide the corresponding genome, this program was excluded in the analysis.

Table 2 shows the predicted results for sRNA Yfr1 and six protein-coding genes. The two som genes PMM1119 and PMM1121 were experimentally validated as *bona fide* targets, and the remaining genes were found to be pseudo targets of Yfr1. As shown in Table 2, sTarPicker predicted two som genes as targets of Yfr1 with probabilities of 1 and 0.837, respectively. The remaining genes were predicted to be pseudo targets as the probabilities were 0.001 or 0. Therefore, the predictions from sTarPicker were in complete agreement with the experimental results. IntaRNA does not provide an obvious indication of interaction, since this method only provides an energy score for each sRNA-target pair. For this reason, the six monocistronic genes with known transcriptional start sites in the top 10 candidates were selected for experimental validation after confining target binding regions to the interval of [−39, +19] in the previous report [38]. Among the six candidate targets for experimental validation, two som genes verified as *bona fide* targets were ranked at positions 1 and 3. The gene PMM0494 verified as a pseudo target was ranked at position 2. Therefore, at lease one false positive was included in the predicted results even if only top three genes were selected as candidate targets. sRNATarget predicted that PMM1119 and PMM0494 were targets of Yfr1 with a probability of 1 and predicted the remaining candidates to be pseudo targets with probabilities less than 0.5. Both a false negative (PMM1121) and a false positive (PMM0494) were identified in the prediction results.

**Table 2:** Predicted results for interaction of Yfr1 and six validated targets.

Target	sTarPicker			IntaRNA		sRNATarget	
	Prob	Rank^a^	Rank^b^	Energy	Rank^b^	Prob	Rank^c^
PMM1119 (som)	1	2	1	−13.42	1	1	1
PMM1121 (som)	0.837	3	2	−10.54	3	0	-
PMM0494 (ppa)	0.001	32	7	−12.58	2	1	1
PMM1697 (σ factor)	0	-	-	−9.03	4	0	-
PMM0538	0	-	-	−8.15	6	0	-
PMM0050 (argJ)	0	-	-	−7.51	10	0	-

The Rank column shows the rank of the target among all predicted targets from the genome-wide prediction. For sTarPicker, results for each sRNA were sorted based on sTarPicker probability (descending), ΔΔG of binding regions (ascending), and ΔΔG of seed regions (ascending). If an sRNA-target pair returned several binding sites, only the site with the smallest rank was selected. For IntaRNA, results were sorted in ascending order of the energies. Only interactions at the interval of [−39, +19] were considered according to their paper. For sRNATarget, results were sorted in descending order of the sRNATarget scores. The first two som genes were experimentally validated and shown to be *bona fide* interaction. The remaining interactions were validated to be non-interaction. ‘-’indicates that no interaction was predicted.

a indicates that the rank was obtained when target binding sites were confined at the interval of [−150, +100].

b indicates that the rank was obtained when target binding sites were confined at the interval of [−39, +19].

c indicates that the rank was obtained with default parameter settings.

Furthermore, sTarPicker only predicted 5 out of 1717 protein-coding genes to be candidate targets with probabilities greater than 0.5 in all annotated protein-coding genes. Two som genes verified as *bona fide* targets were ranked at position 2 and 3 in all 5 candidate targets. Therefore, the PPV for sTarPicker was 0.40 (2/5). The PPV for IntaRNA in the report of Richter et al. [38] was 0.33, since 2 out of 6 candidates were verified as *bona fide* targets. sRNATarget predicted 23 genes to be targets with probabilities greater than 0.5. With a false positive and a false negative, the PPV for sRNATarget is 0.04 (1/23). sTarPicker had the best PPV in the three methods. It should be noted that the six candidate targets for IntaRNA were ranked best only under the following two important circumstances: (1) the sRNA seeds were located in the interval [17], [27], and (2) the target binding sites were located in the interval [−39, +19]. If we confined the target binding site to interval [−39, +19], only two som genes, PMM1119 and PMM1121, were reported to be targets with probabilities greater than 0.5 (File S1). The PPV for sTarPicker increased to 1.00 (2/2), much better than IntaRNA and sRNATarget.

Comparison of binding sites predicted by sTarPicker and IntaRNA showed that they were identical. Taken together, sTarPicker exhibited much better performance than IntaRNA and sRNATarget in the target prediction of sRNA Yfr1.

## Discussion

In this study, we proposed a novel target prediction method, sTarPicker, for prediction of bacterial sRNA targets. The methodology of the program is based on a two-step model of hybridization between an sRNA and a target. In the first step, the sRNA seed binds the target seed by forming a consecutive base-pairing stretch. If the duplex is sufficiently stable, the initial hybrid elongates to form the complete sRNA-target interaction in the second step. Based on the two-step model, sTarPicker first screens seed regions based on an empirical energy value deduced from our training dataset. The program next extends the entire binding site, beginning at the seed regions, mimicking the second step of the model. Through an ensemble classifier trained using the Tclass system, sTarPicker then makes the final prediction regarding whether a sequence represents a target.

Long et al. also employed a two-step model to explore miRNA-target interactions [31]. There are four primary differences between their work and sTarPicker. With respect to the first major difference, Long et al. employed a two-step model for the hybridization between a miRNA and a target. As miRNAs are short, approximately 22 nt in length, they were considered to be unstructured in this hybridization. Only targets were considered to be structured in their model. Therefore, for seed selection, they only considered the stacking energies of miRNA seeds and single-stranded nucleotides of targets. We employed a two-step model for the hybridization between an sRNA and a target. However, in our case, as both sRNAs and targets were long sequences, we considered both to be structured. As a result, we computed the ΔΔG values for seed regions, which included both the total energy of hybridization and the energy required to make both sRNA and target seed regions accessible.

Second, in the model from Long et al., the miRNA nucleates base-pairing with a block of four consecutive unpaired nucleotides in the target in the first step, despite the fact that a longer stretch of base pairs may be present between the miRNA and the target. In contrast, we assumed that the entire sRNA seed combined with the entire target seed in the first step of the model, generating base-paired sequences longer than 4 nt. We do not exclude the possibility that seed base-pairing is initiated by base-pairing between fewer nucleotides than those included in the entire seed length, such that the first step of Long's model becomes a preliminary step for the first step in our model.

Third, Long et al. used a stacking energy value of −4.09 kcal/mol as the initiation threshold, which was based on a previously reported empirical value [43]. The accessibility of target sites was not taken into consideration, since miRNAs were required to bind single-stranded nucleotides within the target. In contrast, we used a ΔΔG value of −2 kcal/mol as the initiation threshold, which was deduced from our training dataset. Accessibility of both sRNA seeds and target seeds were considered in calculation of ΔΔG.

Finally, Long et al. predicted targets according to the total energy for all qualified sites, whereas we constructed an ensemble classifier using machine-learning methods to make final predictions.

In comparison to three state-of-the-art methods for sRNA target prediction, namely IntaRNA, TargetRNA and sRNATarget, sTarPicker performed best in both the accuracy of predicted binding sites and in identification of sRNA targets on an independent test dataset. IntaRNA also required the existence of a seed. However, IntaRNA only added the hybridization energy of seed regions into the extended hybridization energy of the binding sites, and tried to find the hybridization structure with the minimum energy. The site accessibility of seeds was not considered separately in IntaRNA. In sTarPicker, the hybridization energy and site accessibility of seeds were a prerequisite for interaction of two RNA molecules. Only stable hybridized seeds were taken to be extended in the second step of the method. The comparison with IntaRNA showed that the incorporation of site accessibility of seeds and seed selection based on the extended hybridization energy could improve the prediction quality.

Besides IntaRNA, TargetRNA and sRNATarget, we also compared sTarPicker with sRNATargetSVM, the model constructed using SVM in our previous work [17]. To conduct an objective comparison with sRNATargetSVM, we re-trained the model using SVM with the 32 sRNA-target pairs from sTarPicker as positive samples and 64 non-interaction pairs randomly selected from sRNATarBase [12] as negative samples. The core target binding regions were sub-sequences from −150 to +100 nt surrounding the initial start codon and the flanking regions were sub-sequences from −200 to +120 nt surrounding the initial start codon. The detailed methods of sRNATargetSVM2 were provided in File S2. The new model was denoted by sRNATargetSVM2. The sensitivity and specificity of sRNATargetSVM2 on the training data were 87.10% and 100%, respectively. The performance on the training dataset was far better than that of sTarPicker. However, the sensitivity and specificity of sRNATargetSVM2 on the test data were only 35.29% and 95.00%, respectively, which was rather less than those of sTarPicker. The sharp decrease of the performance on the test dataset mainly owed to the use of 10,000 features, because involvement of a large number of features in training models will increase the generalization error in machine learning methods. Moreover, sRNATargetSVM2 employed 10,000 features, whereas sTarPicker employed only five features selected using the Tclass system. The computational cost of sRNATargetSVM2 was about 2,000 times more than that of sTarPicker.

In this study, the training and test datasets included 32 and 17 sRNA-mRNA pairs, respectively. The small numbers of training and test samples owed to the limited number of unique sRNA-mRNA pairs with detailed binding sites experimentally-verified as direct interaction. The small sample size might cause the limited coverage of the whole sRNA-mRNA interaction population, and hence decrease the prediction accuracy. This might be the main reason that all the prediction methods presented by far predict a large number of false positives. With the increase of unique sRNA-mRNA pairs that are experimentally validated to interact directly, the performance of prediction methods would be improved.

Compared to the number of the training samples, the number of features (22) was relatively big. In machine learning methods, the generalization error of the model will be increased if a large number of features are employed to train the model. In this situation, feature subset selection is very important to improve the performance of prediction models. We employed the Tclass system to select a feature subset composed of five features. The performance on the independent test dataset was similar to the performance on the training dataset, suggesting a good generalization ability of sTarPicker.

Currently, sTarPicker was trained and evaluated on one random partition of training and test dataset. To observe the performance of our approach with different partitions of training and test datasets, we randomly selected 32 sRNA-mRNA pairs as a training dataset and the remaining 17 pairs as a positive test dataset. For each sRNA in the positive test dataset, we randomly selected 10 target genes from the corresponding genome as non-interaction targets to compose a negative test dataset. We trained the model using the same steps as those used in sTarPicker and evaluated on the test dataset. The AUC (the Area Under the ROC Curve) value was computed to evaluate the model. The above process was repeated 30 times. All the AUC values for the 30 models were listed in Table S6. The AUC values ranged from 0.8462 to 0.9565. The average was 0.9084. The AUC value for sTarPicker was 0.9164, only slightly higher than the average AUC value. This result demonstrated the robustness of our approach.

### Availability and Future Directions

The genome-wide prediction application is available at http://ccb.bmi.ac.cn/starpicker/prediction.php. The interaction evaluation application is available at http://ccb.bmi.ac.cn/starpicker/evaluation.php.

In the present methodology, we assumed that any part of the sRNA sequences could function as binding sites. However, analysis of all validated binding sites suggested that sRNAs exhibit a strong tendency to bind targets using common regions. For example, *Salmonella* GcvB, an sRNA 201 nt in length, has been shown to interact directly with seven targets via a common region located within 62–92 nt [44]. Therefore, the performance of sTarPicker could be further improved by restricting the functional binding sites of sRNAs.

## Supporting Information

Table S1 sRNA-target pairs used in training.
(DOC)

Table S2 sRNA-target pairs in the test dataset.
(DOC)

Table S3 Comparison of extended and reported binding sites on training dataset.
(DOC)

Table S4 Predicted and validated binding sites on seventeen repression sRNA-target pairs.
(XLS)

Table S5 Predicted results of sTarPicker for six activation sRNA-target pairs.
(DOC)

Table S6 AUC values for thirty models constructed by random partitions of training and test datasets.
(DOC)

File S1 Predicted results of sRNA Yfr1 in *Prochlorococcus* MED4 from sTarPicker.
(TXT)

File S2 Methods for sRNATargetSVM2.
(DOC)
