# Supplementary material for: sTarPicker: A Method for Efficient Prediction of Bacterial sRNA Targets Based on a Two-Step Model for Hybridization
Source: PLoS One. 2011 Jul 22;6(7):e22705. doi: 10.1371/journal.pone.0022705 (PMC3142192; doi:10.1371/journal.pone.0022705)
Supplement: Table S1 — sRNA-target pairs used in training. (DOC) [file pone.0022705.s001.doc]

## Table S1. sRNA-target pairs used in training

| **No** | **Bacterial strain** | **sRNA** | **Target** | **Regulation** |
| --- | --- | --- | --- | --- |
| 1 | Escherichia coli str. K-12 substr. MG1655 | CyaR | luxS | repression |
| 2 | Escherichia coli str. K-12 substr. MG1655 | CyaR | nadE | repression |
| 3 | Escherichia coli str. K-12 substr. MG1655 | CyaR | ompX | repression |
| 4 | Escherichia coli str. K-12 substr. MG1655 | CyaR | yqaE | repression |
| 5 | Escherichia coli str. K-12 substr. MG1655 | DsrA | hns | repression |
| 6 | Escherichia coli str. K-12 substr. MG1655 | GcvB | cycA | repression |
| 7 | Escherichia coli str. K-12 substr. MG1655 | IstR | tisAB | repression |
| 8 | Escherichia coli str. K-12 substr. MG1655 | MicC | ompC | repression |
| 9 | Escherichia coli str. K-12 substr. MG1655 | OxyS | fhlA | repression |
| 10 | Escherichia coli str. K-12 substr. MG1655 | RseX | ompA | repression |
| 11 | Escherichia coli str. K-12 substr. MG1655 | RseX | ompC | repression |
| 12 | Escherichia coli str. K-12 substr. MG1655 | RybB | ompC | repression |
| 13 | Escherichia coli str. K-12 substr. MG1655 | RyhB | fur | repression |
| 14 | Escherichia coli str. K-12 substr. MG1655 | RyhB | iscS | repression |
| 15 | Escherichia coli str. K-12 substr. MG1655 | RyhB | sdhCDAB | repression |
| 16 | Escherichia coli str. K-12 substr. MG1655 | RyhB | sodB | repression |
| 17 | Escherichia coli str. K-12 substr. MG1655 | SgrS | ptsG | repression |
| 18 | Escherichia coli O127:H6 str. E2348/69 | OmrA | cirA | repression |
| 19 | Escherichia coli O127:H6 str. E2348/69 | OmrA | ompR | repression |
| 20 | Escherichia coli O127:H6 str. E2348/69 | OmrA | ompT | repression |
| 21 | Escherichia coli O127:H6 str. E2348/69 | Spot42 | galK | repression |
| 22 | Salmonella enterica subsp. enterica serovar Typhimurium str. LT2 | ChiX | ybfM | repression |
| 23 | Salmonella enterica subsp. enterica serovar Typhimurium str. LT2 | GcvB | argT | repression |
| 24 | Salmonella enterica subsp. enterica serovar Typhimurium str. LT2 | GcvB | dppA | repression |
| 25 | Salmonella enterica subsp. enterica serovar Typhimurium str. LT2 | GcvB | gltI | repression |
| 26 | Salmonella enterica subsp. enterica serovar Typhimurium str. LT2 | GcvB | livJ | repression |
| 27 | Salmonella enterica subsp. enterica serovar Typhimurium str. LT2 | GcvB | STM4351 | repression |
| 28 | Salmonella enterica subsp. enterica serovar Newport str. SL254 | MicA | ompA | repression |
| 29 | Pseudomonas aeruginosa PAO1 | PrrF1 | PA4880 | repression |
| 30 | Staphylococcus aureus subsp. aureus str. MW2 | RNAIII | rot | repression |
| 31 | Staphylococcus aureus subsp. aureus str. NEWMAN | RNAIII | spa | repression |
| 32 | Azotobacter vinelandii DJ | ArrF | fesII | repression |
