# Supplementary material for: sTarPicker: A Method for Efficient Prediction of Bacterial sRNA Targets Based on a Two-Step Model for Hybridization
Source: PLoS One. 2011 Jul 22;6(7):e22705. doi: 10.1371/journal.pone.0022705 (PMC3142192; doi:10.1371/journal.pone.0022705)
Supplement: Table S2 — sRNA-target pairs in the test dataset. (DOC) [file pone.0022705.s002.doc]

## Table S2. sRNA-target pairs in the test dataset

| **No** | **Bacterial strain** | **Accession**  **number** | **Gene**  **number^a^** | **sRNA** | **Target^b^** | **Regulation^c^** | **sTarPicker Probability** |
| --- | --- | --- | --- | --- | --- | --- | --- |
| 1 | Escherichia coli str. K-12 substr. MG1655 | NC_000913 | 4149 | GcvB | sstT | repression | 0.999 |
| 2 | Escherichia coli str. K-12 substr. MG1655 | NC_000913 | 4149 | MicF | ompF | repression | 1.000 |
| 3 | Escherichia coli str. K-12 substr. MG1655 | NC_000913 | 4149 | MicA | phoP | repression | 1.000 |
| 4 | Escherichia coli str. K-12 substr. MG1655 | NC_000913 | 4149 | OmrA | csgD | repression | 1.000 |
| 5 | Salmonella enterica subsp. enterica serovar Typhimurium str. LT2 | NC_003197 | 4423 | GcvB | livK | repression | 1.000 |
| 6 | Salmonella enterica subsp. enterica serovar Typhimurium str. LT2 | NC_003197 | 4423 | GcvB | oppA | repression | 0.857 |
| 7 | Salmonella enterica subsp. enterica serovar Typhimurium str. LT2 | NC_003197 | 4423 | InvR | nmpC | repression | 0.999 |
| 8 | Salmonella enterica subsp. enterica serovar Typhimurium str. LT2 | NC_003197 | 4423 | MicA | lamB | repression | 0.026 |
| 9 | Salmonella enterica subsp. enterica serovar Typhimurium str. LT2 | NC_003197 | 4423 | RybB | ompN | repression | 1.000 |
| 10 | Salmonella enterica subsp. enterica serovar Typhimurium str. LT2 | NC_003197 | 4423 | MicA | ompX | repression | 0.001 |
| 11 | Salmonella enterica subsp. enterica serovar Typhimurium str. LT2 | NC_003197 | 4423 | MicC | nmpC | repression | 0.908 |
| 12 | Pseudomonas aeruginosa PAO1 | NC_002516 | 5566 | PrrF1 | sodB | repression | 0.000 |
| 13 | Vibrio cholerae O1 biovar El Tor str. N16961 | NC_002505  NC_002506 | 3835 | Qrr1 | hapR | repression | 0.768 |
| 14 | Vibrio cholerae O1 biovar eltor str. N16961 | NC_002505  NC_002506 | 3835 | Qrr1 | luxO | repression | 0.560 |
| 15 | Vibrio cholerae O1 biovar eltor str. N16961 | NC_002505  NC_002506 | 3835 | MicX | VC0620 | repression | 1.000 |
| 16 | Vibrio harveyi ATCC BAA-1116 | NC_009783  NC_009784 | 5920 | Qrr1 | luxR | repression | 0.000 |
| 17 | Listeria monocytogenes EGD-e | NC_003210 | 2846 | LhrA | lmo0850 | repression | 0.994 |
| 18 | Escherichia coli str. K-12 substr. MG1655 | NC_000913 | 4149 | GcvB | insH_b2192 | non-interaction | 0.000 |
| 19 | Escherichia coli str. K-12 substr. MG1655 | NC_000913 | 4149 | GcvB | maa_b0459 | non-interaction | 0.000 |
| 20 | Escherichia coli str. K-12 substr. MG1655 | NC_000913 | 4149 | GcvB | mog_b0009 | non-interaction | 0.000 |
| 21 | Escherichia coli str. K-12 substr. MG1655 | NC_000913 | 4149 | GcvB | rhaD_b3902 | non-interaction | 0.225 |
| 22 | Escherichia coli str. K-12 substr. MG1655 | NC_000913 | 4149 | GcvB | gabP_b2663 | non-interaction | 0.001 |
| 23 | Escherichia coli str. K-12 substr. MG1655 | NC_000913 | 4149 | GcvB | ycgK_b1178 | non-interaction | 0.080 |
| 24 | Escherichia coli str. K-12 substr. MG1655 | NC_000913 | 4149 | GcvB | insH_b1370 | non-interaction | 0.000 |
| 25 | Escherichia coli str. K-12 substr. MG1655 | NC_000913 | 4149 | GcvB | ykfB_b0250 | non-interaction | 0.000 |
| 26 | Escherichia coli str. K-12 substr. MG1655 | NC_000913 | 4149 | GcvB | dsbD_b4136 | non-interaction | 0.000 |
| 27 | Escherichia coli str. K-12 substr. MG1655 | NC_000913 | 4149 | GcvB | recG_b3652 | non-interaction | 0.192 |
| 28 | Escherichia coli str. K-12 substr. MG1655 | NC_000913 | 4149 | MicF | rfaY_b3625 | non-interaction | 0.000 |
| 29 | Escherichia coli str. K-12 substr. MG1655 | NC_000913 | 4149 | MicF | znuA_b1857 | non-interaction | 0.002 |
| 30 | Escherichia coli str. K-12 substr. MG1655 | NC_000913 | 4149 | MicF | ldhA_b1380 | non-interaction | 0.000 |
| 31 | Escherichia coli str. K-12 substr. MG1655 | NC_000913 | 4149 | MicF | alsC_b4086 | non-interaction | 0.000 |
| 32 | Escherichia coli str. K-12 substr. MG1655 | NC_000913 | 4149 | MicF | infA_b0884 | non-interaction | 0.000 |
| 33 | Escherichia coli str. K-12 substr. MG1655 | NC_000913 | 4149 | MicF | rof_b0189 | non-interaction | 0.000 |
| 34 | Escherichia coli str. K-12 substr. MG1655 | NC_000913 | 4149 | MicF | relE_b1563 | non-interaction | 0.000 |
| 35 | Escherichia coli str. K-12 substr. MG1655 | NC_000913 | 4149 | MicF | arpA_b4017 | non-interaction | 0.000 |
| 36 | Escherichia coli str. K-12 substr. MG1655 | NC_000913 | 4149 | MicF | fimI_b4315 | non-interaction | 0.000 |
| 37 | Escherichia coli str. K-12 substr. MG1655 | NC_000913 | 4149 | MicF | uhpT_b3666 | non-interaction | 0.000 |
| 38 | Escherichia coli str. K-12 substr. MG1655 | NC_000913 | 4149 | MicA | bcp_b2480 | non-interaction | 0.000 |
| 39 | Escherichia coli str. K-12 substr. MG1655 | NC_000913 | 4149 | MicA | fabA_b0954 | non-interaction | 0.000 |
| 40 | Escherichia coli str. K-12 substr. MG1655 | NC_000913 | 4149 | MicA | dctA_b3528 | non-interaction | 0.000 |
| 41 | Escherichia coli str. K-12 substr. MG1655 | NC_000913 | 4149 | MicA | malZ_b0403 | non-interaction | 0.000 |
| 42 | Escherichia coli str. K-12 substr. MG1655 | NC_000913 | 4149 | MicA | rfbC_b2038 | non-interaction | 0.000 |
| 43 | Escherichia coli str. K-12 substr. MG1655 | NC_000913 | 4149 | MicA | sanA_b2144 | non-interaction | 0.000 |
| 44 | Escherichia coli str. K-12 substr. MG1655 | NC_000913 | 4149 | MicA | osmB_b1283 | non-interaction | 0.000 |
| 45 | Escherichia coli str. K-12 substr. MG1655 | NC_000913 | 4149 | MicA | rfbB_b2041 | non-interaction | 0.000 |
| 46 | Escherichia coli str. K-12 substr. MG1655 | NC_000913 | 4149 | MicA | murG_b0090 | non-interaction | 0.025 |
| 47 | Escherichia coli str. K-12 substr. MG1655 | NC_000913 | 4149 | MicA | xisE_b1141 | non-interaction | 0.000 |
| 48 | Escherichia coli str. K-12 substr. MG1655 | NC_000913 | 4149 | OmrA | dld_b2133 | non-interaction | 0.004 |
| 49 | Escherichia coli str. K-12 substr. MG1655 | NC_000913 | 4149 | OmrA | ynaK_b1365 | non-interaction | 0.000 |
| 50 | Escherichia coli str. K-12 substr. MG1655 | NC_000913 | 4149 | OmrA | solA_b1059 | non-interaction | 0.000 |
| 51 | Escherichia coli str. K-12 substr. MG1655 | NC_000913 | 4149 | OmrA | clpP_b0437 | non-interaction | 0.000 |
| 52 | Escherichia coli str. K-12 substr. MG1655 | NC_000913 | 4149 | OmrA | lptC_b3199 | non-interaction | 0.000 |
| 53 | Escherichia coli str. K-12 substr. MG1655 | NC_000913 | 4149 | OmrA | flgD_b1075 | non-interaction | 0.000 |
| 54 | Escherichia coli str. K-12 substr. MG1655 | NC_000913 | 4149 | OmrA | flgE_b1076 | non-interaction | 0.000 |
| 55 | Escherichia coli str. K-12 substr. MG1655 | NC_000913 | 4149 | OmrA | pstS_b3728 | non-interaction | 0.000 |
| 56 | Escherichia coli str. K-12 substr. MG1655 | NC_000913 | 4149 | OmrA | actP_b4067 | non-interaction | 0.155 |
| 57 | Escherichia coli str. K-12 substr. MG1655 | NC_000913 | 4149 | OmrA | ymiA_b4522 | non-interaction | 0.000 |
| 58 | Salmonella enterica subsp. enterica serovar Typhimurium str. LT2 | NC_003197 | 4423 | GcvB | ygiY_STM3178 | non-interaction | 0.000 |
| 59 | Salmonella enterica subsp. enterica serovar Typhimurium str. LT2 | NC_003197 | 4423 | GcvB | proY_STM0400 | non-interaction | 0.000 |
| 60 | Salmonella enterica subsp. enterica serovar Typhimurium str. LT2 | NC_003197 | 4423 | GcvB | rpmD_STM3422 | non-interaction | 0.000 |
| 61 | Salmonella enterica subsp. enterica serovar Typhimurium str. LT2 | NC_003197 | 4423 | GcvB | bcp_STM2491 | non-interaction | 0.001 |
| 62 | Salmonella enterica subsp. enterica serovar Typhimurium str. LT2 | NC_003197 | 4423 | GcvB | emtA_STM1799 | non-interaction | 0.000 |
| 63 | Salmonella enterica subsp. enterica serovar Typhimurium str. LT2 | NC_003197 | 4423 | GcvB | ydeV_STM4072 | non-interaction | 0.000 |
| 64 | Salmonella enterica subsp. enterica serovar Typhimurium str. LT2 | NC_003197 | 4423 | GcvB | entB_STM0597 | non-interaction | 0.000 |
| 65 | Salmonella enterica subsp. enterica serovar Typhimurium str. LT2 | NC_003197 | 4423 | GcvB | phoU_STM3853 | non-interaction | 0.000 |
| 66 | Salmonella enterica subsp. enterica serovar Typhimurium str. LT2 | NC_003197 | 4423 | GcvB | yoaA_STM1821 | non-interaction | 0.245 |
| 67 | Salmonella enterica subsp. enterica serovar Typhimurium str. LT2 | NC_003197 | 4423 | GcvB | yeaS_STM1270 | non-interaction | 0.002 |
| 68 | Salmonella enterica subsp. enterica serovar Typhimurium str. LT2 | NC_003197 | 4423 | InvR | modB_STM0782 | non-interaction | 0.000 |
| 69 | Salmonella enterica subsp. enterica serovar Typhimurium str. LT2 | NC_003197 | 4423 | InvR | yraO_STM3266 | non-interaction | 0.000 |
| 70 | Salmonella enterica subsp. enterica serovar Typhimurium str. LT2 | NC_003197 | 4423 | InvR | coaD_STM3725 | non-interaction | 0.060 |
| 71 | Salmonella enterica subsp. enterica serovar Typhimurium str. LT2 | NC_003197 | 4423 | InvR | STM4212 | non-interaction | 0.000 |
| 72 | Salmonella enterica subsp. enterica serovar Typhimurium str. LT2 | NC_003197 | 4423 | InvR | mdoG_STM1150 | non-interaction | 0.000 |
| 73 | Salmonella enterica subsp. enterica serovar Typhimurium str. LT2 | NC_003197 | 4423 | InvR | pnuC_STM0757 | non-interaction | 0.000 |
| 74 | Salmonella enterica subsp. enterica serovar Typhimurium str. LT2 | NC_003197 | 4423 | InvR | STM2377 | non-interaction | 0.000 |
| 75 | Salmonella enterica subsp. enterica serovar Typhimurium str. LT2 | NC_003197 | 4423 | InvR | xni_STM2972 | non-interaction | 0.000 |
| 76 | Salmonella enterica subsp. enterica serovar Typhimurium str. LT2 | NC_003197 | 4423 | InvR | parC_STM3174 | non-interaction | 0.000 |
| 77 | Salmonella enterica subsp. enterica serovar Typhimurium str. LT2 | NC_003197 | 4423 | InvR | tilS_STM0236 | non-interaction | 0.000 |
| 78 | Salmonella enterica subsp. enterica serovar Typhimurium str. LT2 | NC_003197 | 4423 | MicA | dnaG_STM3210 | non-interaction | 0.000 |
| 79 | Salmonella enterica subsp. enterica serovar Typhimurium str. LT2 | NC_003197 | 4423 | MicA | iclR_STM4187 | non-interaction | 0.000 |
| 80 | Salmonella enterica subsp. enterica serovar Typhimurium str. LT2 | NC_003197 | 4423 | MicA | rhaR_STM4049 | non-interaction | 0.000 |
| 81 | Salmonella enterica subsp. enterica serovar Typhimurium str. LT2 | NC_003197 | 4423 | MicA | murC_STM0129 | non-interaction | 0.000 |
| 82 | Salmonella enterica subsp. enterica serovar Typhimurium str. LT2 | NC_003197 | 4423 | MicA | STM3160 | non-interaction | 0.000 |
| 83 | Salmonella enterica subsp. enterica serovar Typhimurium str. LT2 | NC_003197 | 4423 | MicA | sinH_STM2517 | non-interaction | 0.000 |
| 84 | Salmonella enterica subsp. enterica serovar Typhimurium str. LT2 | NC_003197 | 4423 | MicA | menE_STM2305 | non-interaction | 0.000 |
| 85 | Salmonella enterica subsp. enterica serovar Typhimurium str. LT2 | NC_003197 | 4423 | MicA | rlpA_STM0638 | non-interaction | 0.000 |
| 86 | Salmonella enterica subsp. enterica serovar Typhimurium str. LT2 | NC_003197 | 4423 | MicA | gltI_STM0665 | non-interaction | 0.000 |
| 87 | Salmonella enterica subsp. enterica serovar Typhimurium str. LT2 | NC_003197 | 4423 | MicA | ytfE_STM4399 | non-interaction | 0.000 |
| 88 | Salmonella enterica subsp. enterica serovar Typhimurium str. LT2 | NC_003197 | 4423 | RybB | yieM_STM3878.S | non-interaction | 0.000 |
| 89 | Salmonella enterica subsp. enterica serovar Typhimurium str. LT2 | NC_003197 | 4423 | RybB | acrD_STM2481 | non-interaction | 0.000 |
| 90 | Salmonella enterica subsp. enterica serovar Typhimurium str. LT2 | NC_003197 | 4423 | RybB | hsdS_STM4524 | non-interaction | 0.000 |
| 91 | Salmonella enterica subsp. enterica serovar Typhimurium str. LT2 | NC_003197 | 4423 | RybB | STM1472 | non-interaction | 0.000 |
| 92 | Salmonella enterica subsp. enterica serovar Typhimurium str. LT2 | NC_003197 | 4423 | RybB | rplU_STM3304 | non-interaction | 0.000 |
| 93 | Salmonella enterica subsp. enterica serovar Typhimurium str. LT2 | NC_003197 | 4423 | RybB | umuD_STM1998 | non-interaction | 0.000 |
| 94 | Salmonella enterica subsp. enterica serovar Typhimurium str. LT2 | NC_003197 | 4423 | RybB | yfiA_STM2665 | non-interaction | 0.000 |
| 95 | Salmonella enterica subsp. enterica serovar Typhimurium str. LT2 | NC_003197 | 4423 | RybB | STM1005 | non-interaction | 0.000 |
| 96 | Salmonella enterica subsp. enterica serovar Typhimurium str. LT2 | NC_003197 | 4423 | RybB | fabF_STM1197 | non-interaction | 0.000 |
| 97 | Salmonella enterica subsp. enterica serovar Typhimurium str. LT2 | NC_003197 | 4423 | RybB | fhuB_STM0194 | non-interaction | 0.000 |
| 98 | Salmonella enterica subsp. enterica serovar Typhimurium str. LT2 | NC_003197 | 4423 | MicC | ung_STM2647 | non-interaction | 0.000 |
| 99 | Salmonella enterica subsp. enterica serovar Typhimurium str. LT2 | NC_003197 | 4423 | MicC | STM0327 | non-interaction | 0.000 |
| 100 | Salmonella enterica subsp. enterica serovar Typhimurium str. LT2 | NC_003197 | 4423 | MicC | rpmC_STM3432 | non-interaction | 0.000 |
| 101 | Salmonella enterica subsp. enterica serovar Typhimurium str. LT2 | NC_003197 | 4423 | MicC | priA_STM4095 | non-interaction | 0.000 |
| 102 | Salmonella enterica subsp. enterica serovar Typhimurium str. LT2 | NC_003197 | 4423 | MicC | ydjN_STM1320 | non-interaction | 0.000 |
| 103 | Salmonella enterica subsp. enterica serovar Typhimurium str. LT2 | NC_003197 | 4423 | MicC | STM0353 | non-interaction | 0.000 |
| 104 | Salmonella enterica subsp. enterica serovar Typhimurium str. LT2 | NC_003197 | 4423 | MicC | queA_STM0404 | non-interaction | 0.000 |
| 105 | Salmonella enterica subsp. enterica serovar Typhimurium str. LT2 | NC_003197 | 4423 | MicC | yfcB_STM2385 | non-interaction | 0.000 |
| 106 | Salmonella enterica subsp. enterica serovar Typhimurium str. LT2 | NC_003197 | 4423 | MicC | pta_STM2338 | non-interaction | 0.000 |
| 107 | Salmonella enterica subsp. enterica serovar Typhimurium str. LT2 | NC_003197 | 4423 | MicC | yrdA_STM3399 | non-interaction | 0.000 |
| 108 | Pseudomonas aeruginosa PAO1 | NC_002516 | 5566 | PrrF1 | PA1031 | non-interaction | 0.000 |
| 109 | Pseudomonas aeruginosa PAO1 | NC_002516 | 5566 | PrrF1 | PA5393 | non-interaction | 0.000 |
| 110 | Pseudomonas aeruginosa PAO1 | NC_002516 | 5566 | PrrF1 | PA2776 | non-interaction | 0.000 |
| 111 | Pseudomonas aeruginosa PAO1 | NC_002516 | 5566 | PrrF1 | PA4673 | non-interaction | 0.011 |
| 112 | Pseudomonas aeruginosa PAO1 | NC_002516 | 5566 | PrrF1 | PA0680 | non-interaction | 0.165 |
| 113 | Pseudomonas aeruginosa PAO1 | NC_002516 | 5566 | PrrF1 | PA5532 | non-interaction | 0.014 |
| 114 | Pseudomonas aeruginosa PAO1 | NC_002516 | 5566 | PrrF1 | PA2206 | non-interaction | 0.000 |
| 115 | Pseudomonas aeruginosa PAO1 | NC_002516 | 5566 | PrrF1 | yajC_PA3822 | non-interaction | 0.000 |
| 116 | Pseudomonas aeruginosa PAO1 | NC_002516 | 5566 | PrrF1 | PA3712 | non-interaction | 0.000 |
| 117 | Pseudomonas aeruginosa PAO1 | NC_002516 | 5566 | PrrF1 | hasE_PA3405 | non-interaction | 0.000 |
| 118 | Vibrio cholerae O1 biovar El Tor str. N16961 | NC_002505  NC_002506 | 3835 | Qrr1 | aspA_VC2698 | non-interaction | 0.019 |
| 119 | Vibrio cholerae O1 biovar El Tor str. N16961 | NC_002505  NC_002506 | 3835 | Qrr1 | VC2300 | non-interaction | 0.000 |
| 120 | Vibrio cholerae O1 biovar El Tor str. N16961 | NC_002505  NC_002506 | 3835 | Qrr1 | VC1456 | non-interaction | 0.000 |
| 121 | Vibrio cholerae O1 biovar El Tor str. N16961 | NC_002505  NC_002506 | 3835 | Qrr1 | sspA_VC0576 | non-interaction | 0.000 |
| 122 | Vibrio cholerae O1 biovar El Tor str. N16961 | NC_002505  NC_002506 | 3835 | Qrr1 | VC1358 | non-interaction | 0.000 |
| 123 | Vibrio cholerae O1 biovar El Tor str. N16961 | NC_002505  NC_002506 | 3835 | Qrr1 | VC1189 | non-interaction | 0.003 |
| 124 | Vibrio cholerae O1 biovar El Tor str. N16961 | NC_002505  NC_002506 | 3835 | Qrr1 | hemE_VC0332 | non-interaction | 0.000 |
| 125 | Vibrio cholerae O1 biovar El Tor str. N16961 | NC_002505  NC_002506 | 3835 | Qrr1 | VC0438 | non-interaction | 0.000 |
| 126 | Vibrio cholerae O1 biovar El Tor str. N16961 | NC_002505  NC_002506 | 3835 | Qrr1 | VC0508 | non-interaction | 0.000 |
| 127 | Vibrio cholerae O1 biovar El Tor str. N16961 | NC_002505  NC_002506 | 3835 | Qrr1 | VC0069 | non-interaction | 0.000 |
| 128 | Vibrio cholerae O1 biovar eltor str. N16961 | NC_002505  NC_002506 | 3835 | MicX | VC1988 | non-interaction | 0.000 |
| 129 | Vibrio cholerae O1 biovar eltor str. N16961 | NC_002505  NC_002506 | 3835 | MicX | VC2531 | non-interaction | 0.000 |
| 130 | Vibrio cholerae O1 biovar eltor str. N16961 | NC_002505  NC_002506 | 3835 | MicX | VC1148 | non-interaction | 0.000 |
| 131 | Vibrio cholerae O1 biovar eltor str. N16961 | NC_002505  NC_002506 | 3835 | MicX | VC1839 | non-interaction | 0.000 |
| 132 | Vibrio cholerae O1 biovar eltor str. N16961 | NC_002505  NC_002506 | 3835 | MicX | VC1051 | non-interaction | 0.996 |
| 133 | Vibrio cholerae O1 biovar eltor str. N16961 | NC_002505  NC_002506 | 3835 | MicX | rpmD_VC2578 | non-interaction | 0.151 |
| 134 | Vibrio cholerae O1 biovar eltor str. N16961 | NC_002505  NC_002506 | 3835 | MicX | VC0889 | non-interaction | 0.003 |
| 135 | Vibrio cholerae O1 biovar eltor str. N16961 | NC_002505  NC_002506 | 3835 | MicX | ilvM_VC0030 | non-interaction | 0.006 |
| 136 | Vibrio cholerae O1 biovar eltor str. N16961 | NC_002505  NC_002506 | 3835 | MicX | VC2648 | non-interaction | 0.005 |
| 137 | Vibrio cholerae O1 biovar eltor str. N16961 | NC_002505  NC_002506 | 3835 | MicX | VC0472 | non-interaction | 0.000 |
| 138 | Vibrio harveyi ATCC BAA-1116 | NC_009783  NC_009784 | 5920 | Qrr1 | VIBHAR_03358 | non-interaction | 0.000 |
| 139 | Vibrio harveyi ATCC BAA-1116 | NC_009783  NC_009784 | 5920 | Qrr1 | VIBHAR_03644 | non-interaction | 0.000 |
| 140 | Vibrio harveyi ATCC BAA-1116 | NC_009783  NC_009784 | 5920 | Qrr1 | VIBHAR_00024 | non-interaction | 0.000 |
| 141 | Vibrio harveyi ATCC BAA-1116 | NC_009783  NC_009784 | 5920 | Qrr1 | VIBHAR_02793 | non-interaction | 0.000 |
| 142 | Vibrio harveyi ATCC BAA-1116 | NC_009783  NC_009784 | 5920 | Qrr1 | VIBHAR_01080 | non-interaction | 0.000 |
| 143 | Vibrio harveyi ATCC BAA-1116 | NC_009783  NC_009784 | 5920 | Qrr1 | VIBHAR_01541 | non-interaction | 0.002 |
| 144 | Vibrio harveyi ATCC BAA-1116 | NC_009783  NC_009784 | 5920 | Qrr1 | VIBHAR_02569 | non-interaction | 0.000 |
| 145 | Vibrio harveyi ATCC BAA-1116 | NC_009783  NC_009784 | 5920 | Qrr1 | VIBHAR_02787 | non-interaction | 0.000 |
| 146 | Vibrio harveyi ATCC BAA-1116 | NC_009783  NC_009784 | 5920 | Qrr1 | VIBHAR_01100 | non-interaction | 0.000 |
| 147 | Vibrio harveyi ATCC BAA-1116 | NC_009783  NC_009784 | 5920 | Qrr1 | VIBHAR_02627 | non-interaction | 0.000 |
| 148 | Listeria monocytogenes EGD-e | NC_003210 | 2846 | LhrA | lmo1620 | non-interaction | 0.030 |
| 149 | Listeria monocytogenes EGD-e | NC_003210 | 2846 | LhrA | folK_lmo0226 | non-interaction | 0.000 |
| 150 | Listeria monocytogenes EGD-e | NC_003210 | 2846 | LhrA | lmo0593 | non-interaction | 0.000 |
| 151 | Listeria monocytogenes EGD-e | NC_003210 | 2846 | LhrA | lmo0429 | non-interaction | 0.000 |
| 152 | Listeria monocytogenes EGD-e | NC_003210 | 2846 | LhrA | lmo2065 | non-interaction | 0.000 |
| 153 | Listeria monocytogenes EGD-e | NC_003210 | 2846 | LhrA | lmo2238 | non-interaction | 0.000 |
| 154 | Listeria monocytogenes EGD-e | NC_003210 | 2846 | LhrA | radC_lmo1549 | non-interaction | 0.001 |
| 155 | Listeria monocytogenes EGD-e | NC_003210 | 2846 | LhrA | lmo0689 | non-interaction | 0.000 |
| 156 | Listeria monocytogenes EGD-e | NC_003210 | 2846 | LhrA | lmo0326 | non-interaction | 0.000 |
| 157 | Listeria monocytogenes EGD-e | NC_003210 | 2846 | LhrA | lmo1494 | non-interaction | 0.000 |

^a^The column of gene number shows the total protein-coding gene number of the bacterial genome described in ptt file downloaded from GenBank. ^b^Target name for non-interaction target includes gene name and its synonym, linked with an underline. The synonym is a unique identifier for a gene in the ptt file. If the gene has no gene name, only a synonym is included. ^c^Regulation column includes two types of regulation. The regulation type of 17 validated sRNA-target pairs is repression. The regulation type of the remaining 140 pairs is non-interaction, since these pairs were obtained by randomly selecting 10 protein-coding genes for each sRNA.
