# Supplementary material for: sTarPicker: A Method for Efficient Prediction of Bacterial sRNA Targets Based on a Two-Step Model for Hybridization
Source: PLoS One. 2011 Jul 22;6(7):e22705. doi: 10.1371/journal.pone.0022705 (PMC3142192; doi:10.1371/journal.pone.0022705)
Supplement: Table S3 — Comparison of extended and reported binding sites on training dataset. (DOC) [file pone.0022705.s003.doc]

## Table S3. Comparison of extended and reported binding sites on training dataset

| **No** | **Bacterial strain** | **sRNA-target pair** | **Extended binding site** | | **Reported binding site** | | **Ratio I^c^** | **Ratio II^d^** |
| --- | --- | --- | --- | --- | --- | --- | --- | --- |
|  |  |  | **sRNA^a^** | **Target^b^** | **sRNA^a^** | **Target^b^** |  |  |
| 1 | Escherichia coli str. K-12 substr. MG1655 | CyaR-luxS | 26..49 | -12..13 | 35..49 | -12..3 | 0.612 | 1.000 |
| 2 | Escherichia coli str. K-12 substr. MG1655 | CyaR-ompX | 36..48 | -9..3 | 38..48 | -9..2 | 0.880 | 1.000 |
| 3 | Escherichia coli str. K-12 substr. MG1655 | CyaR-yqaE | 35..43 | 4..12 | 31..50 | -4..16 | 1.000 | 0.450 |
| 4 | Escherichia coli str. K-12 substr. MG1655 | DsrA-hns | 31..60 | -10..19 | 31..43 | 7..19 | 0.441 | 1.000 |
| 5 | Escherichia coli str. K-12 substr. MG1655 | GcvB-cycA | 124..161 | -26..7 | 124..161 | -26..7 | 1.000 | 1.000 |
| 6 | Escherichia coli str. K-12 substr. MG1655 | IstR-tisAB | 65..85 | -122..-102 | 57..95 | -135..-92 | 1.000 | 0.506 |
| 7 | Escherichia coli str. K-12 substr. MG1655 | MicC-ompC | 1..16 | -30..-15 | 1..30 | -41..-15 | 1.000 | 0.561 |
| 8 | Escherichia coli str. K-12 substr. MG1655 | OxyS-fhlA | 81..104 | -15..7 | 98..104 | -15..-9 | 0.304 | 1.000 |
| 9 | Escherichia coli str. K-12 substr. MG1655 | RseX-ompA | 42..49 | -21..-14 | 37..50 | -22..-8 | 1.000 | 0.552 |
| 10 | Escherichia coli str. K-12 substr. MG1655 | RseX-ompC | 30..43 | -14..-1 | 30..55 | -31..-1 | 1.000 | 0.491 |
| 11 | Escherichia coli str. K-12 substr. MG1655 | RybB-ompC | 46..49^f^ | -45..-42^f^ | 1..30, 46..57 | -53..-4 | 1.000 | 0.087 |
| 12 | Escherichia coli str. K-12 substr. MG1655 | RyhB-fur | 38..47 | -60..-51 | 38..76 | -96..-47 | 1.000 | 0.225 |
| 13 | Escherichia coli str. K-12 substr. MG1655 | RyhB-iscS | 40..69 | -27..3 | 43..68 | -26..-1 | 0.867 | 1.000 |
| 14 | Escherichia coli str. K-12 substr. MG1655 | RyhB-sdhCDAB | 12..40 | -32..-6 | 9..50 | -42..-3 | 1.000 | 0.683 |
| 15 | Escherichia coli str. K-12 substr. MG1655 | RyhB-sodB | 38..46 | -4..5 | 38..56 | -12..5 | 1.000 | 0.500 |
| 16 | Escherichia coli str. K-12 substr. MG1655 | SgrS-ptsG | 170..186 | -27..-9 | 157..187 | -28..4 | 1.000 | 0.571 |
| 17 | Escherichia coli O127:H6 str. E2348/69 | OmrA-cirA | 2..10 | -19..-10 | 2..24 | -35..-10 | 1.000 | 0.388 |
| 18 | Escherichia coli O127:H6 str. E2348/69 | OmrA-ompR | 1..9 | -19..-11 | 1..19 | -29..-11 | 1.000 | 0.474 |
| 19 | Escherichia coli O127:H6 str. E2348/69 | OmrA-ompT | 1..13 | 8..20 | 1..33 | -12..20 | 1.000 | 0.400 |
| 20 | Escherichia coli O127:H6 str. E2348/69 | Spot42-galK | 8..48 | -9..31 | 1..62 | -20..56 | 1.000 | 0.587 |
| 21 | Salmonella enterica subsp. enterica serovar Typhimurium str. LT2 | ChiX-ybfM | 42..53 | -19..-8 | 42..53 | -19..-8 | 1.000 | 1.000 |
| 22 | Salmonella enterica subsp. enterica serovar Typhimurium str. LT2 | GcvB-argT | 70..91 | -57..-37 | 70..91 | -57..-37 | 1.000 | 1.000 |
| 23 | Salmonella enterica subsp. enterica serovar Typhimurium str. LT2 | GcvB-dppA | 57..90 | -34..-7 | 65..82 | -30..-14 | 0.565 | 1.000 |
| 24 | Salmonella enterica subsp. enterica serovar Typhimurium str. LT2 | GcvB-gltI | 64..76 | -38..-26 | 66..76 | -38..-27 | 0.885 | 1.000 |
| 25 | Salmonella enterica subsp. enterica serovar Typhimurium str. LT2 | GcvB-livJ | 63..91 | -62..-28 | 63..87 | -51..-28 | 0.766 | 1.000 |
| 26 | Salmonella enterica subsp. enterica serovar Typhimurium str. LT2 | GcvB-STM4351 | 66..77 | -30..-20 | 62..87 | -41..-16 | 1.000 | 0.442 |
| 27 | Salmonella enterica subsp. enterica serovar Newport str. SL254 | MicA-ompA | 9..25 | 4..19 | 9..25 | 4..19 | 1.000 | 1.000 |
| 28 | Pseudomonas aeruginosa PAO1 | PrrF1-PA4880 | 96..119 | -27..-7 | 84..118 | -26..7 | 0.956 | 0.632 |
| 29 | Staphylococcus aureus subsp. aureus str. MW2 | RNAIII-rot | 239..259 | 31..50 | 229..269 | 23..61 | 1.000 | 0.513 |
| 30 | Staphylococcus aureus subsp. aureus str. NEWMAN | RNAIII-spa | 408..433 | 25..46 | 384..445 | 13..69 | 1.000 | 0.403 |
| 31 | Azotobacter vinelandii DJ | ArrF-FeSII | 91..107 | -39..-24 | 88..107 | -41..-8 | 1.000 | 0.611 |

In 32 training sRNA-target pairs, only 31 pairs had qualified seed regions. Therefore, binding sites from these 31 seed regions were extended. The reported binding sites were partially validated by experiments. Some reported binding sites were predicted according to the rough binding regions validated by experiments.

^a^The column of sRNA shows the start and stop position of binding sites, delimited by double dot.

^b^The column of Target shows the start and stop position of binding sites relative to the start codon, delimited by double dot.

^c^Ratio I is the ratio of overlapped nucleotides between extended and reported binding sites to extended binding sites. It equals to the positive predictive value (PPV).

^d^Ratio II is the ratio of overlapped nucleotides between extended and reported binding sites to reported binding sites. It equals to the sensitivity.

^f^In this special case, the binding sites were only 4nt in length. However, we checked the seed of this interaction and found that the seed was 6nt in length. The terminal two nucleotides formed intra-molecular pairing during the process of binding site extension. Therefore, the length of binding sites was shorter than the seed length.
