# Supplementary material for: sTarPicker: A Method for Efficient Prediction of Bacterial sRNA Targets Based on a Two-Step Model for Hybridization
Source: PLoS One. 2011 Jul 22;6(7):e22705. doi: 10.1371/journal.pone.0022705 (PMC3142192; doi:10.1371/journal.pone.0022705)
Supplement: Table S5 — Predicted results of sTarPicker for six activation sRNA-target pairs. (DOC) [file pone.0022705.s005.doc]

## Table S5. Predicted results of sTarPicker for six activation sRNA-target pairs

| **No** | **Bacterial strain** | **Accession number** | **Protein-coding gene number** | **sRNA** | **Target** | **Regulation** | **sTarPicker Probability** |
| --- | --- | --- | --- | --- | --- | --- | --- |
| 1 | Escherichia coli str. K-12 substr. MG1655 | NC_000913 | 4149 | DsrA | rpoS | activation | 1.000 |
| 2 | Escherichia coli str. K-12 substr. MG1655 | NC_000913 | 4149 | RprA | rpoS | activation | 1.000 |
| 3 | Escherichia coli str. K-12 substr. MG1655 | NC_000913 | 4149 | RyhB | shiA | activation | 0 |
| 4 | Escherichia coli str. K-12 substr. MG1655 | NC_000913 | 4149 | GlmZ | glmS | activation | 0.645 |
| 5 | Azotobacter vinelandii DJ | NC_012560 | 5051 | ArrF | phbR | activation | 0 |
| 6 | Azotobacter vinelandii DJ | NC_012560 | 5051 | ArrF | sodB | activation | 0.009 |

The column of Protein-coding gene number shows the total gene number of the bacterial genome.
