# Supplementary material for: sTarPicker: A Method for Efficient Prediction of Bacterial sRNA Targets Based on a Two-Step Model for Hybridization
Source: PLoS One. 2011 Jul 22;6(7):e22705. doi: 10.1371/journal.pone.0022705 (PMC3142192; doi:10.1371/journal.pone.0022705)
Supplement: Table S6 — AUC values for thirty models constructed by random partitions of training and test datasets. (DOC) [file pone.0022705.s006.doc]

## Table S6. AUC values for thirty models constructed by random partitions of training and test datasets

| Model | AUC value | Model | AUC value | Model | AUC value |
| --- | --- | --- | --- | --- | --- |
| 1 | 0.8719 | 11 | 0.9431 | 21 | 0.8923 |
| 2 | 0.8715 | 12 | 0.9342 | 22 | 0.8576 |
| 3 | 0.8681 | 13 | 0.9317 | 23 | 0.9168 |
| 4 | 0.9348 | 14 | 0.8801 | 24 | 0.9253 |
| 5 | 0.9293 | 15 | 0.8635 | 25 | 0.9416 |
| 6 | 0.9333 | 16 | 0.9267 | 26 | 0.9283 |
| 7 | 0.9132 | 17 | 0.9565 | 27 | 0.8845 |
| 8 | 0.8659 | 18 | 0.9533 | 28 | 0.9095 |
| 9 | 0.8760 | 19 | 0.9389 | 29 | 0.9231 |
| 10 | 0.8462 | 20 | 0.9255 | 30 | 0.9088 |
