# Supplementary material for: sTarPicker: A Method for Efficient Prediction of Bacterial sRNA Targets Based on a Two-Step Model for Hybridization
Source: PLoS One. 2011 Jul 22;6(7):e22705. doi: 10.1371/journal.pone.0022705 (PMC3142192; doi:10.1371/journal.pone.0022705)
Supplement: File S2 — Methods for sRNATargetSVM2. (DOC) [file pone.0022705.s008.doc]

## Methods for sRNATargetSVM2

The flanking sequences around the core binding regions were extracted using sliding windows. For each sub-sequence, ten features, including the percent composition of bases in interior loops, the minimum free energy (MFE) of hybridization, and the difference in the MFE values before and after hybridization, were calculated. The model was then trained using support vector machines (SVM).

The libSVM package (version 2.84) [1] was employed. The optimal penalty parameter *C* and the radial basis function (RBF) kernel parameter γ were found using the grid search strategy provided in the package. The search space for *C* and γ were both [2^-10^, 2^10^] and the search step was 2^1^. The 5-fold cross validation accuracy was used as the objective function. After grid search, the optimal penalty parameter *C* 1.0 and the optimal RBF kernel parameter γ 0.0009765625 were obtained.

**References**

1. Chang C-C, Lin C-J. LIBSVM: a library for support vector machine. 2001
